# Expression Pattern of Sonic Hedgehog, Patched and Smoothened in Clear Cell Renal Carcinoma

**DOI:** 10.3390/ijms24108935

**Published:** 2023-05-18

**Authors:** Ana Dunatov Huljev, Nela Kelam, Benjamin Benzon, Violeta Šoljić, Natalija Filipović, Valdi Pešutić Pisac, Merica Glavina Durdov, Katarina Vukojević

**Affiliations:** 1 Department of Pathology, Forensic Medicine and Cytology, University Hospital of Split, 21000 Split, Croatia; adunatov@kbsplit.hr (A.D.H.); valdypp@gmail.com (V.P.P.); merigdst@yahoo.co.uk (M.G.D.); 2 Department of Anatomy, Histology and Embryology, University of Split School of Medicine, 21000 Split, Croatia; nela.kelam@mefst.hr (N.K.); benjamin.benzon@mefst.hr (B.B.); natalija.filipovic@mefst.hr (N.F.); 3 Department of Anatomy, Histology and Embryology, School of Medicine, University of Mostar, 88000 Mostar, Bosnia and Herzegovina; violeta.soljic@mef.sum.ba; 4 Faculty of Health Studies, University of Mostar, 88000 Mostar, Bosnia and Herzegovina; 5 Center for Translational Research in Biomedicine, University of Split School of Medicine, 21000 Split, Croatia

**Keywords:** renal cell carcinoma, Sonic Hedgehog, patched, smoothened

## Abstract

Clear cell renal cell carcinoma (ccRCC) is the deadliest neoplasm of the urinary tract, and we are still far from completely understanding ccRCC development and treatment. The renal tissue paraffin blocks (20) of patients with ccRCC were collected at the University Hospital in Split from 2019 to 2020, and tissue sections were stained with patched (PTCH), anti-smoothened (SMO) and anti-Sonic Hedgehog (SHH) antibodies. SHH was highly expressed (31.9%) in grade 1 tumour, it being higher than all other grades and the control (*p* < 0.001–*p* < 0.0001). The trend of a linear decrease in the expression of SHH was observed with the progression of the tumour grade (*p* < 0.0001). PTCH expression was significantly lower in grades 1 and 2 in comparison to the control (*p* < 0.01) and grade 4 (*p* < 0.0001). A significant increase in the expression of SMO was found in grade 4 compared to all other grades (*p* < 0.0001) and the control (*p* < 0.001). The strong expression of SHH was observed in carcinoma cells of the G1 stage with a diffuse staining pattern (>50% of neoplastic cells). Stroma and/or inflammatory infiltrate display no staining and no expression of SHH in G1 and G2, while mild focal staining (10–50% of neoplastic cells) was observed in G3 and G4. Patients with high PTCH and low SMO expression had significant time survival differences (*p* = 0.0005 and *p* = 0.029, respectively). Therefore, high levels of PTCH and low levels of SMO expression are important markers of better survival rates in ccRCC patients.

## 1. Introduction

Renal cell carcinoma is the seventh and tenth most common cancer in men and women. It is the 16th most common cause of death, and among urinary tract neoplasms, it is the deadliest [1]. Throughout history, different morphological subtypes have been described regarding cytological or architectural features (e.g., clear cell renal cell carcinoma—ccRCC, papillary RCC, chromophobe RCC), and with the advancement of molecular techniques, new, molecularly defined entities have emerged (e.g., TFE3-rearranged renal cell carcinoma, succinate dehydrogenase-deficient renal cell carcinoma, ALK-rearranged renal cell carcinoma) [2]. ccRCC is the most common renal cell carcinoma (60–75%) [1]. It is a morphologically heterogeneous tumour derived from tubular epithelial cells, composed of cells with clear or eosinophilic cytoplasm. Often, it occurs sporadically, rarely in hereditary settings (e.g., von Hippel–Lindau syndrome, Constitutional chromosome 3 translocation, Cowden syndrome, Birt–Hogg–Dube syndrome) [3]. Risk factors are obesity, smoking, hypertension, acquired cystic kidney disease, exposure to trichloroethylene and genetic susceptibility [4]. Prognostic factors are pathological stage, tumour grade, presence of tumour necrosis and rhabdoid or sarcomatous differentiation. Today, four-tiered WHO/ISUP grading is used based on nucleolar prominence, nuclear pleomorphism and/or rhabdoid and/or sarcomatoid differentiation [5]. In pancreatic and oesophageal cancer, Hh is activated in the early stages and in metastasis [6]. In gastric and prostate cancer, it is activated in tissue invasion and increased metastatic potential [7]. Treatment for CRCC depends on TNM classification and includes surgery, immunotherapy and targeted therapy. Thermal ablation and cryosurgery for smaller tumour treatment modalities such as radiation therapy are used. However, the existence of different carcinogens and variable genetic predisposition makes it difficult to determine key factors in the development of ccRCC [8].

**Figure 1:**
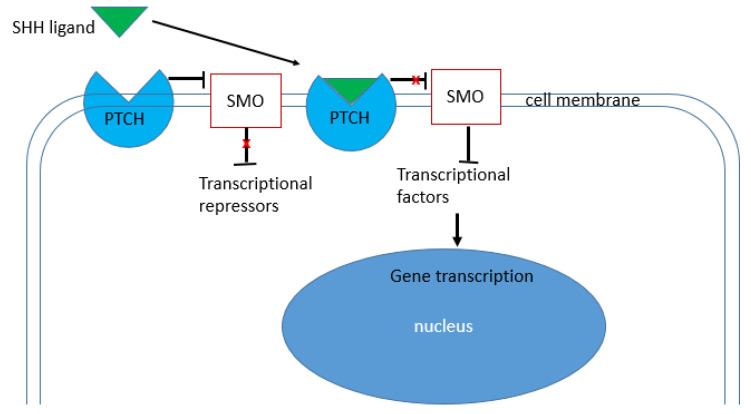
The figure depicts a simple overview of the SHH signalling pathway. When the SHH ligand is absent, PTCH activate SMO, and there is no gene transcription (red cross). When the SHH ligand binds to the PTCH protein, PTCH is inhibited and leads to SMO inhibition (red cross), which then leads to gene transcription.

The Hedgehog (Hh) signalling pathway is an evolutionary conserved pathway that has a significant role in normal embryonic development. Nevertheless, this pathway can be reactivated in wound healing, tissue repair, cell regeneration and carcinogenesis [8]. In Hh signalling activation, three proteins are involved: Hedgehog (HH) Ligand, Patched (PTCH) and Smoothened (SMO). Humans have three *Hh genes*: *Sonic Hedgehog* (*Shh*), *Indian Hedgehog* (*Ihh*) and *Desert Hedgehog* (*Dhh*). Proteins derived from these genes bind to the Ptch receptor protein on the cell membrane, relieving Smo inhibition and allowing the Hh signal to be transmitted downstream in a phosphorylation cascade to Gli proteins (Figure 1). Without the Hh ligand, GliFl is phosphorylated into a GliR form that translocates to the nucleus, binds to Hh target gene promoters and inhibits their expression [9]. The binding of the Hh ligand leads to the formation of GliA. GliA then migrates to the nucleus, binds to target gene promoters and activates the transcription of Hh target genes [10].

The *Sonic* (*SHH*) gene encodes a protein essential in patterning the early embryo; it acts as the instrumental inductive signal patterning nervous system and limbs. Defects in this protein or its signalling pathway are a cause of holoprosencephaly (HPE) [11] and VACTERL syndrome (vertebral defects, anal atresia, cardiac defects, trachea-esophageal fistula, renal anomalies and limb abnormalities). In adults, SHH helps to control stem cell proliferation. It is typically expressed in the foetal intestine, liver, lung and kidney but not in adult tissues [12]. Many cancers use the Sonic Hedgehog pathway: small cell lung carcinoma, basal cell carcinoma, medulloblastoma, glioblastoma as well as breast, liver, colon, pancreatic and prostate cancers [13,14]. In endometrial cancer, it has favourable prognostic use.

Patched (PTCH) acts as a receptor for Sonic Hedgehog (SHH), Indian Hedgehog (IHh) and Desert Hedgehog (DHh), and together with Smoothened protein (SMO), trans-duces Hedgehog’s protein signal [15]. It seems to have a tumour suppression function. Cytoplasmic expression is seen in many tissues, both regular and tumourous [15].

Smoothened (SMO) acts as a transducing subunit and is normally inhibited by PTCH. With Hh binding to PTCH, that inhibition is relieved, allowing the Hh signal to be transmitted downstream to Gli proteins. The role of SMO has been investigated in many cancers: breast, liver, pancreas and colon [16]. Its overexpression is associated with tumour size, invasiveness, metastasis and recurrence [17]. In addition, SMO inhibitors reduce cell proliferation and trigger apoptosis [18].

So far, dysfunction of the Hh signalling pathway has been described in some developmental deformities and cancer, such as basal cell carcinoma, medulloblastoma, neuroblastoma and ccRCC, by using different molecular techniques [10,19]. These data supported the development of new therapeutical modalities targeting the SHH signalling pathway, such as inhibitors of SMO, cyclopamine [20] and a combination of the GLIs inhibitor Gant61 and the AKT inhibitor Perifosine [21,22,23]. However, the results of the expression of the Hedgehog signalling pathway in ccRCC are inconsistent. One study reported increased SHH gene expression in ccRCC at the mRNA and protein levels [24], while others reported lower levels of SHH mRNA [25]. To our knowledge, double immunofluorescence analysis has not been used for this purpose. Therefore, we decided to perform this analysis on Hh pathway proteins in order to see whether increased expression can be seen by this method and if there is any difference between tumourous and nontumourous (normal) kidney tissue. Even though significant advances in understanding molecular processes in renal cell carcinomas have been made, we are still far from thoroughly understanding RCC development, let alone from being able to cure advanced renal cancer.

## 2. Results

**Figure 2:**
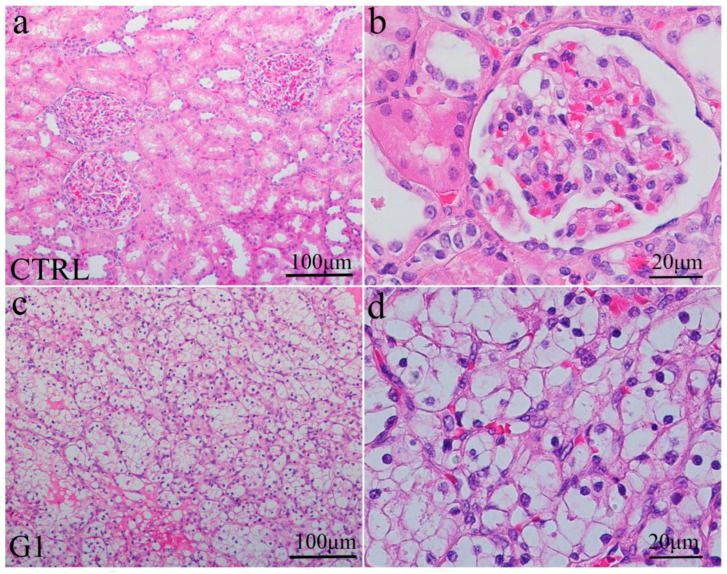

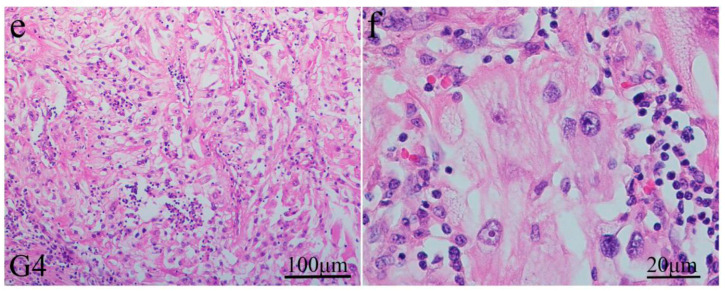
Hematoxylin and eosin staining of normal kidney cortex (CTRL) and different grades of renal clear cell carcinoma (ccRCC). Normal glomeruli and tubules (**a**); higher magnification of glomeruli and tubules in the renal cortex (**b**); clear cell carcinoma gradus 1—G1 (**c**); higher magnification of ccRCC gradus 1 (**d**); ccRCC gradus 4—G4 (**e**) and higher magnification of ccRCC gradus 4 (**f**). Scale bars indicated on panels.

Clear cell renal carcinoma (ccRCC) originates from epithelial cells of proximal tubules. Different grades of ccRCC were studied. Grade 1 is characterised by small nuclei with inconspicuous nucleoli, while the highest grade (grade 4) is characterised by nuclear polymorphism and rhabdoid or sarcomatoid features (Figure 2).

**Figure 3:**
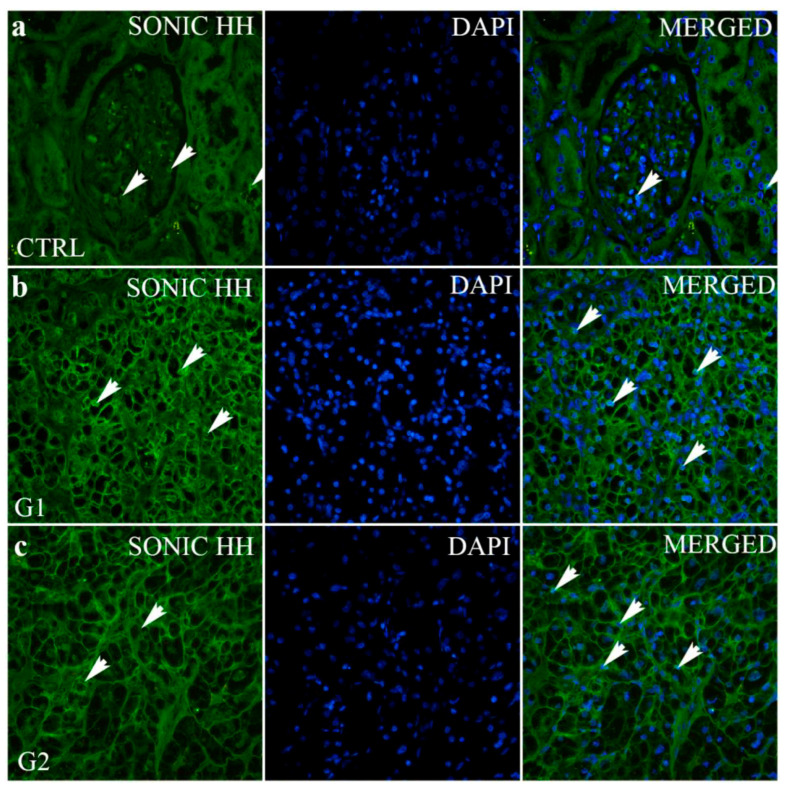

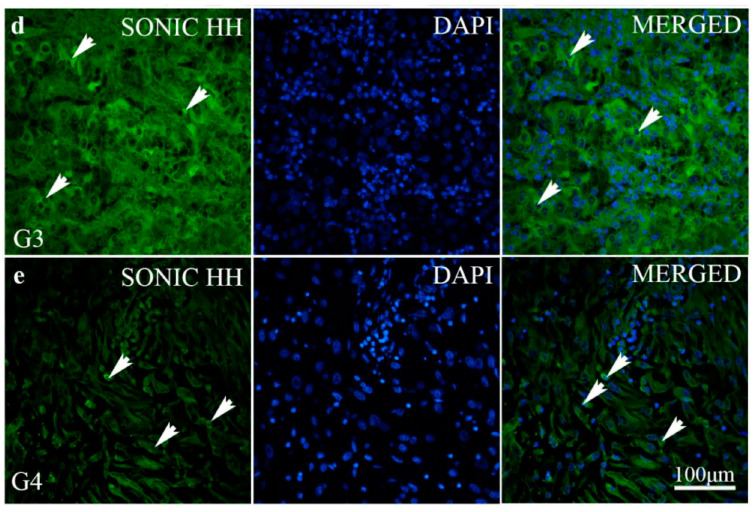
Sonic hedgehog (SHH) immunofluorescence staining of a normal kidney cortex (CTRL) and different grades of renal clear cell carcinoma (ccRCC). Normal glomeruli and tubules (**a**); ccRCC gradus 1—G1 (**b**); ccRCC gradus 2—G2 (**c**); ccRCC gradus 3—G3 (**d**) and ccRCC gradus 4—G4 (**e**). SHH positive cells (arrows—positive cells regardless of staining intensity).

In the renal cortex of normal healthy kidneys, Sonic HH is expressed occasionally in both glomeruli and tubules (Figure 3).

**Figure 4:**
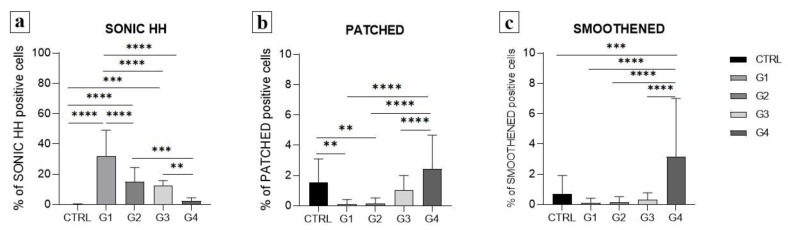
Graphic presentation of quantitative expression of Sonic Hedgehog (**a**), patched (**b**) and smoothened (**c**) in the control (CTRL) and different grades of ccRCC (G1–G4). Data are presented as mean ± SD. ** *p* < 0.01, *** *p* < 0.001, **** *p* < 0.0001, one-way ANOVA followed by Tukey’s multiple comparison test.

On the contrary, Sonic HH is highly expressed in grade 1 tumour (Figure 3), with 31.9% of positive cells (Figure 4). The highest expression of SHH was observed in grade 1, it being higher than all other grades and the control (*p* < 0.001 to *p* < 0.0001). The trend of a linear decrease in the expression of SHH was observed with the progression of the tumour grade (*p* < 0.0001).

**Figure 5:**
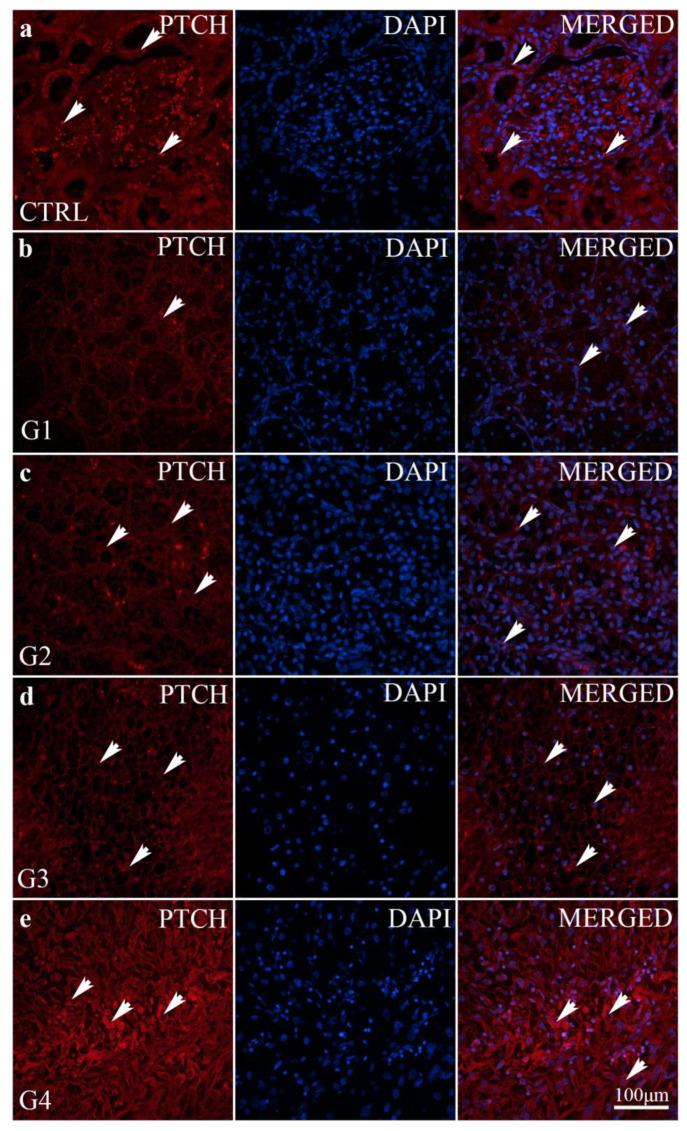
Patched (PTCH) immunofluorescence staining of a normal kidney cortex (CTRL) and different grades of ccRCC. Normal glomeruli and tubules (**a**); ccRCC gradus 1—G1 (**b**); ccRCC gradus 2—G2 (**c**); ccRCC gradus 3—G3 (**d**) and ccRCC gradus 4—G4 (**e**). PTCH positive cells (arrows—positive cells regardless of staining intensity).

PTCH has a low expression pattern in all samples, including the control (Figure 5). There is significantly lower PTCH expression in grades 1 and 2 in comparison to the control (*p* < 0.01) and grade 4 (*p* < 0.0001). The PTCH expression in grades 3 and 4 did not significantly differ from that of the control samples (*p* > 0.05) (Figure 4).

**Figure 6:**
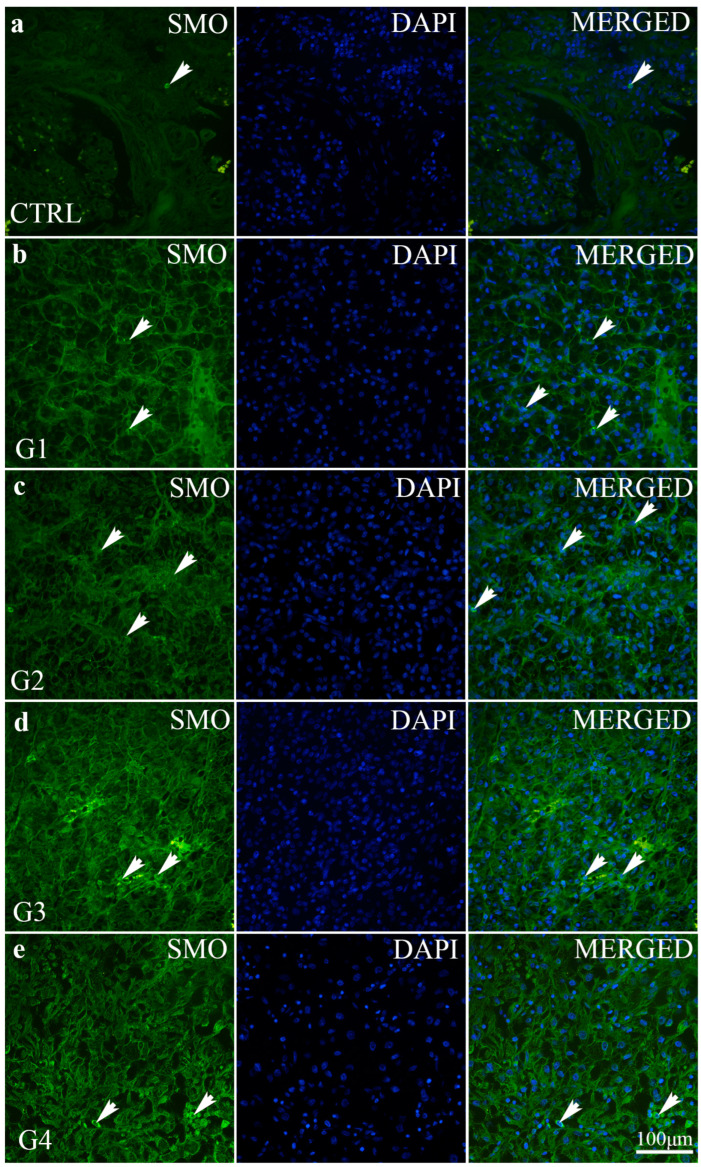
Smoothened (SMO) immunofluorescence staining of a normal kidney cortex (CTRL) and different grades of ccRCC. Normal glomeruli and tubules (**a**); ccRCC gradus 1—G1 (**b**); ccRCC gradus 2—G2 (**c**); ccRCC gradus 3—G3 (**d**) and ccRCC gradus 4—G4 (**e**). SMO positive cells (arrows—positive cells regardless of staining intensity).

Similar to PTCH, the expression of SMO is also very low (under 10% of positive cells) (Figure 6). However, a significant increase in the expression of SMO was found in grade 4 in comparison to all other grades (*p* < 0.0001) and the control (*p* < 0.001) (Figure 4).

We observed differences in the intensity and distribution of staining for SHH, PTCH and SMO in different grades of ccRCC and the control kidney. All investigated markers display no staining or expression in the control kidney, except for the mild expression of SHH in epithelial cells (Table 1). The strong expression of SHH was observed in carcinoma cells of the G1 stage of ccRCC with a diffuse staining pattern (>50% of neoplastic cells), while SHH expression was mild in carcinoma cells of the G4 stage of ccRCC with less than 10% of stained carcinoma cells. Stroma and/or inflammatory infiltrate display no staining and no expression of SHH in the G1 and G2 stages of ccRCC, while mild focal staining (10–50% of neoplastic cells) was observed in G3 and G4 of ccRCC. PTCH and SMO display no staining and no expression in the control kidney and G1–G3 stages of ccRCC, while in the G4 stage of ccRCC, moderate and focal PTCH and SMO staining expression was seen (Table 1).

**Figure 7:**
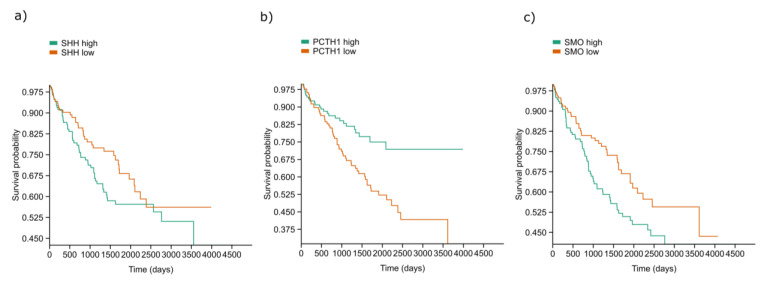
Graphic presentation of survival analysis (days) of SHH (**a**), PTCH (**b**) and SMO (**c**) in high (green line) and low (red line) mRNA expression in ccRCC was expressed as the average survival time in days. The KM method and log rank test for survival length were used. Data are used from the GDC TCGA Kidney Clear Cell Carcinoma (KIRC) study.

The SHH, PTCH and SMO in high and low protein expressions in ccRCC were analysed for the survival rate, and the average survival time was calculated (Figure 7). Both SHH high and low expressions displayed no statistically significant survival time differences (*p* = 0.1095). SHH high expression had an average survival time of 1309.8 days, and SHH low expression had an average survival time of 1314.6 days. However, considering PTHC and SMO expression, we observed significant time survival differences with ccRCC patients with a high expression of PTCH and a low expression of SMO (*p* = 0.0005 and *p* = 0.029, respectively). PTCH high expression had an average survival time of 1302.1 days, while PTHC low expression had an average survival time of 1284.7 days. SMO high expression had an average survival time of 1236.7 days, and SMO low expression had an average survival time of 1380.4. days.

## 3. Discussion

Dysregulation of the Hh signalling pathway was thought to lead to its irregular activation, which can result in malignant transformation through changes in cell behaviour mechanisms such as stimulating the target genes transcription and adjusting the cell cycle. These mechanisms are initiated through three proteins: Sonic Hedgehog, Patched and Smoothened. Sonic Hedgehog serves as a ligand, Patched serves as a membrane receptor and Smoothened serves as a co-receptor [26]. The characteristic of the Patched membrane receptor is that it is not activated by ligand binding but is suppressed by this interaction [26].

In our study, in the renal cortex of a normal healthy kidney, Sonic HH is expressed occasionally in both glomeruli and tubules, which is in correlation with other studies that found no expression of SHH in adult normal tissue. On the contrary, Sonic HH is highly expressed in about one-third of the cells in grade 1 ccRCC. This HH expression might imply the activation of the HH signalling pathway that is usually only expressed in fetal development and numerous types of cancer. A high expression of SHH was accompanied by a low expression of Patched in the G1 grade, while in grade G4, the opposite is true. The HH signalling through the positive feedback of its signalling molecules as targeting genes (PTCH1 and GLI1) is self-amplified, and the activated cells produce more ligands under an oncogenic status [10]. However, there are no differences in the survival rate of ccRCC patients, regardless of whether SHH expression is low or high.

In our study, the expression of the SHH protein did not follow the expression of SMO. There might be speculation about possible genetic mutation in the activation of SHH pathways, known for the basal cell nevus or Gorlin syndrome, which predisposes patients to the early development of multiple BCCs [27]. Therefore, the negative feedback loop of Patch on SHH signalling is not in line, which might contribute to an improper SHH signalling pathway. In support of this, Kim et al. reported that the increased expression of Ptch1, Ptch2 and Gli1 genes could serve as a very reliable indicator of SHH signalling pathway activation, ensuring negative (Ptch1) and positive (Gli1) regulation by a feedback mechanism [27]. The PTCH1 receptor inhibits Sonic Hedgehog signalling when it is unliganded [28]. Additionally, although PTCH expression was deficient in G1 and G3 grades, it is interesting that the ratio between SHH and PTCH is high in grade G4 of ccRCC. This finding is in line with the fact that the increased expression of PTCH is a reliable indicator of an activated SHH signalling pathway. This is also in line with a negative feedback loop of PTCH1 to the SHH pathway. Kabaria et al. provided data on many factors that were related to the patient’s survival [4]. The factors included the tumour grading, the tumour stage, the nutrition status, the patient’s ECOG (Eastern Cooperative Oncology Group) performance status and so on [29]. However, a limitation of our study is that we just checked the different expression levels of SHH, PTCH and SMO in different tumour gradings, and then we used analysis data from the GDC TCGA renal cell carcinoma (282 patients) to draw the survival curve. However, our findings regarding the survival might be caused by other factors (e.g., ECOG score, TMN stage, chemotherapy side-effects) besides investigated proteins.

Transcriptomic data (used from the UCSCI Xena database) suggest that the high expression of PTCH contributes to a higher survival rate of ccRCC patients. Kotulak-Chrzaszcz et al. found high mRNA levels of SHH, SMO and GLI1-3 in various tumour tissue, especially in the early ccRCC [23]. Additionally, they performed Cyclopamine treatment in vitro and revealed that Cyclopamine arrests 786-O cells in the G2/M phase and decreases the expression levels of GLI1. However, although we can rely on SHH signalling proteins as biomarkers not only in ccRCC but in a pan-cancer model as well, we should be aware that treatment modalities require verification by further in vivo studies.

In our study, low levels of PTCH in our samples suggest a poor prognosis for these patients. Our results are in line with studies performed by Zhou et al. and Kotulak-Chrzaszcz, who found a considerable decrease in the PTCH1 mRNA level in ccRCC [24,25].

In the G1 grade, ccRCC is highly differentiated, and in the G4 grade, the tumour is poorly differentiated. In our study, in the G4 stage of ccRCC, the morphological appearance of the tumour displayed carcinoma cells that look like mesenchymal cells or sarcomatoid cells. This finding might indicate an epithelial–mesenchymal transition. In our study, PTCH and SMO expression in the G4 stage of ccRCC was increased in stromal cells instead of cancer cells. However, this finding might be explained by epithelial–mesenchymal transition and indicates that these cells are, in fact, tumour cells. This finding also implies that the SHH pathway is not working correctly. Smo expression is mostly shown in stromal cells rather than in tumour cells in the G4 grade. This finding might also be related to paracrine signalisation, as shown in colon cancer, prostate cancer and pancreas cancer [10]. Our results revealed a statistically significant increase in the Smo expression in the G4 grade of ccRCC, compared to control tissues. Additionally, the Smo staining pattern in the G4 stage of ccRCC is displayed in the whole cytoplasm, which might be in line with the lower expression of SHH. On the contrary, the membrane pattern of staining implies that SMO is in the correct position for activation. This finding is also in line with the study performed by Kotulak-Chrzaszcz et al. [24] and implies the necessity of using the Smo as a potential drug target for ccRCC since low levels of Smo provide a better survival rate.

PTCH has a low expression pattern in all samples, including the control. There is significantly lower PTCH expression in grades 1 and 2 of ccRCC compared to the control and grade 4. The PTCH expression in grades 3 and 4 of ccRCC did not significantly differ from that of control samples. Similar to PTCH, the expression of SMO is also deficient (under 10% of positive cells). However, the significant increase in the expression of SMO was found in grade 4 compared to all other grades and the control. Interestingly, in all tumour samples, with G4 being the only exception, there was an increase in SMO, and it is only expressed in G4. This supports the finding that drugs (Vismodegib) used for the treatment of BCC and glioblastoma need to be carefully considered in patients with the G4 grade of ccRCC [26]. Additionally, the substance acts as a cyclopamine-competitive antagonist of the smoothened receptor (SMO), part of the Hedgehog signalling pathway [30]. SMO inhibition causes the transcription factors GLI1 and GLI2 to remain inactive, preventing tumour-mediating genes’ expression within the Hedgehog pathway. This pathway is pathogenetically relevant in more than 90% of basal-cell carcinomas [31].

In conclusion, patients with the G4 grade have higher SMO expression, especially in stromal cells, implying the worst survival rate. This group of patients might benefit the most from SMO inhibitors. On the other hand, in grade G1 of ccRCC, SHH had the highest expression in cancer cells, which might imply its role in the early carcinogenesis and impaired signalling of subsequent genes involved in this pathway. Therefore, gene expression-based studies provide valuable data for setting prognostic signatures and a more precise assessment of the individual risk of the progression of ccRCC patients and better disease management.

## 4. Materials and Methods

### 4.1. Tissue Procurement and Processing

The sample includes 20 patients with a pathological diagnosis of ccRCC (Table 2). Renal tissue paraffin blocks from nephrectomy were collected at the Department of Pathology, Forensic Medicine and Cytology at the University Hospital of Split from 2019 to 2020 and processed with the permission of the Ethical Committee of the University Hospital in Split (class: 500-03/23-01/04, ur. broj:2181-147/01/06/LJ.Z.-23-02), following the Helsinki Declaration. Clinical data from the time of the biopsy were extracted from the pathology report. Inclusion criteria required sufficient paraffin block material for immunohistochemistry (IHC) and complete clinical data. Exclusion criteria comprised incomplete laboratory results or insufficient material for IHC.

Kidney tissue samples were removed and separately fixed with 4% paraformaldehyde (PFA) in 0.1 M phosphate buffer saline (PBS) overnight for conventional histological analyses (hematoxylin-eosin and immunofluorescence staining). Following tissue fixation and dehydration using graded ethanol solutions, the tissue was embedded in paraffin blocks, serially cut to a thickness of 4 µm and then mounted on glass slides.

### 4.2. Immunofluorescence

After deparaffinisation in xylol and rehydration in graded ethanol solutions, mounted tissue samples were cooked in a water steamer in the 0.01 M citrate buffer (pH 6.0) for 30 min at 95 °C and cooled to room temperature. After washing with 0.1 M PBS, a protein-blocking solution (ab64226, Abcam, Cambridge, UK) was used for 20 min to prevent nonspecific staining. Sections were covered with primary antibodies (Table 2) and incubated overnight in a humidity chamber. The next day, slides were rinsed with PBS before incubation with secondary antibodies (Table 3) for one hour. DAPI (4′,6-diamidino-2-phenylindole) staining was applied to visualise nuclei, following a final PBS wash of the samples. The samples were air-dried and slip-covered (Immuno-Mount, Thermo Shandon, Pittsburgh, PA, USA).

In the preadsorption test, each primary antibody was diluted to the prescribed concentration in the blocking solution. Then, a solution containing a suitable peptide antigen was applied to tissue sections. There was no evidence of antibody staining. No nonspecific secondary antibody binding or false-positive results were identified when primary antibodies were excluded from the immunofluorescence procedure.

### 4.3. Data Acquisition and Statistical Analysis of the Area Percentage

Images for (H&E) tissue sections were collected using a light microscope (BX40, Olympus, Tokyo, Japan). In addition, images of the kidney cortex were captured by an immunofluorescence microscope (BX51, Olympus, Tokyo, Japan) upgraded with a Nikon DS-Ri2 camera (Nikon Corporation, Tokyo, Japan) with NIS-Elements F software version 3.0. To assess the immunoexpression of the Sonic HH, Smoothened and Patched, ten non-overlapping representative visual fields were captured at ×40 magnification and constant exposure times and analysed. Green staining, diffuse or punctate, was interpreted as positive for Sonic HH and Smoothened, while red was considered positive for the expression of Patched.

Semiquantitative analysis was performed for all three markers, Sonic HH, Smoothened and Patched, with the staining intensity labelled as: (−) no expression; (+) mild expression; (++) moderate expression; (+++) strong expression. In addition, the extension of staining was categorised as: (n) no staining (<10% of epithelial/carcinoma cells); (f) focal staining (10–50% of neoplastic cells) and (d) diffuse staining (>50% of neoplastic cells).

All acquired images were processed with ImageJ software version 1.51 (NIH, Bethesda, MD, USA) for quantitative cell immunoreactivity evaluation. Figures for area percentage analysis were prepared using the subtraction of the median filter and colour thresholding to measure the section percentage area covered by the positive signal, as described previously [32,33]. Three expert histologists set background thresholds using negative control images. Statistical analyses were performed using GraphPad Prism 9.0.0 (GraphPad Software, San Diego, CA, USA), with the probability level of *p* < 0.05 regarded as statistically significant. The percentage of positive cells was presented as the mean ± standard deviation (SD). All graphs were assembled using GraphPad Prism 9.0.0. Plates were created using Adobe Photoshop (Adobe, San Jose, CA, USA).

Considering interobserver variability, three researchers independently examined the collected microphotographs. Interrater agreement was demonstrated by interclass correlation analysis, which provided a coefficient greater than 0.80, suggesting excellent agreement [34]. Survival analysis was carried out by the KM method and log rank test, and the survival length was expressed as the average survival time.

### 4.4. Transcriptomics

We exported the data for the RNA expression of the *SHH*, *PTCH1* and *SMO* genes from the UCSC Xena database (University of California Santa Cruz, http://xena.ucsc.edu/ (accessed on 4 February 2023)). Overall survival and expression of SHH, PTHC and SMO (mRNA seq) data from the GDC TCGA Kidney Clear Cell Carcinoma (KIRC) study was downloaded and processed in Microsoft Excel (Microsoft Corp., Redmond, WA, USA). Overall, 282 patient samples were included in the survival analysis after data curation for double samples (Figure 7). Survival analysis based on expression quartiles, i.e., between the lowest and highest quartile for each gene, was conducted in Past 4.0 software [35]. For statistical analysis, the Kaplan Meier method and log rank test were used for survival length analysis.

## Figures and Tables

**Table 1:** Intensity and extension of staining for Sonic HH, Patched and Smoothened in different grades of ccRCC (G1–G4) and the control kidney (CTRL).

		Sonic HH	Patched	Smoothened
CTRL	epithelial cells	+/n	−/n	−/n
	Stroma/inflammatory infiltrate	−/n	−/n	−/n
G1	carcinoma cells	+++/d	−/n	−/n
	Stroma/inflammatory infiltrate	−/n	−/n	−/n
G2	carcinoma cells	++/f	−/n	−/n
	Stroma/inflammatory infiltrate	−/n	−/n	−/n
G3	carcinoma cells	++/f	−/n	−/n
	Stroma/inflammatory infiltrate	+/f	−/n	−/n
G4	carcinoma cells	+/n	−/n	−/n
	Stroma/inflammatory infiltrate	+/f	++/f	++/f

− no expression; + mild expression; ++ moderate expression; +++ strong expression; n no staining (<10% of epithelial/carcinoma cells); f focal staining (10–50% of neoplastic cells); d diffuse staining (>50% of neoplastic cells).

**Table 2:** Patients’ characteristics according to ccRCC grades.

Hystology and Grade		No. Patients (n)	Age (Year)	Male/Female (n)
ccRCC	Grade G1	5	64 ± 11	3/2
	Grade G2	5	63 ± 6	3/2
	Grade G3	5	65 ± 7	4/1
	Grade G4	5	64 ± 8	4/1

Values are means ± standard deviations.

**Table 3:** Antibodies used for immunofluorescence.

Antibodies		Catalogue Number	Host	Dilution	Source
Primary	Patched (G-19)	sc-6149	Goat	1:200	Santa Cruz Bt. (Texas, TX, USA)
	Anti-Smoothened antibody	ab72130	Rabbit	1:100	Abcam (Cambridge, UK)
	Anti-Sonic Hedgehog antibody (5H4)	ab135240	Mouse	1:200	Abcam (Cambridge, UK)
Secondary	Anti-Goat IgG, Alexa Fluor^®^ 594	705-295-003	Donkey	1:400	Jackson Immuno Research Laboratories, Inc. (Baltimore, PA, USA)
	Anti-Rabbit IgG, Alexa Fluor^®^ 488	711-545-152	Donkey	1:400	Jackson Immuno Research Laboratories, Inc. (Baltimore, PA, USA)
	Anti-Mouse IgG, Alexa Fluor^®^ 488	715-545-150	Donkey	1:400	Jackson Immuno ResearchLaboratories, Inc. (Baltimore, PA, USA)

## Data Availability

All data and materials are available upon request.

## References

[1] Makino T., Kadomoto S., Izumi K., Mizokami A. (2022). Epidemiology and Prevention of Renal Cell Carcinoma. Cancers.

[2] Lobo J., Ohashi R., Helmchen B.M., Rupp N.J., Ruschoff J.H., Moch H. (2021). The Morphological Spectrum of Papillary Renal Cell Carcinoma and Prevalence of Provisional/Emerging Renal Tumor Entities with Papillary Growth. Biomedicines.

[3] Trpkov K., Hes O., Williamson S.R., Adeniran A.J., Agaimy A., Alaghehbandan R., Amin M.B., Argani P., Chen Y.B., Cheng L. (2021). New developments in existing WHO entities and evolving molecular concepts: The Genitourinary Pathology Society (GUPS) update on renal neoplasia. Mod. Pathol. Off. J. United States Can. Acad. Pathol. Inc..

[4] Kabaria R., Klaassen Z., Terris M.K. (2016). Renal cell carcinoma: Links and risks. Int. J. Nephrol. Renov. Dis..

[5] Liu N., Gan W., Qu F., Wang Z., Zhuang W., Agizamhan S., Xu L., Yin J., Guo H., Li D. (2018). Does the Fuhrman or World Health Organization/International Society of Urological Pathology Grading System Apply to the Xp11.2 Translocation Renal Cell Carcinoma?: A 10-Year Single-Center Study. Am. J. Pathol..

[6] Liu H., Zhao Y.R., Chen B., Ge Z., Huang J.S. (2019). High expression of SMARCE1 predicts poor prognosis and promotes cell growth and metastasis in gastric cancer. Cancer Manag. Res..

[7] Xu Y., Song S., Wang Z., Ajani J.A. (2019). The role of hedgehog signaling in gastric cancer: Molecular mechanisms, clinical potential, and perspective. Cell Commun. Signal. CCS.

[8] Jonasch E., Walker C.L., Rathmell W.K. (2021). Clear cell renal cell carcinoma ontogeny and mechanisms of lethality. Nat. Rev. Nephrol..

[9] Qi X., Li X. (2020). Mechanistic Insights into the Generation and Transduction of Hedgehog Signaling. Trends Biochem. Sci..

[10] Skoda A.M., Simovic D., Karin V., Kardum V., Vranic S., Serman L. (2018). The role of the Hedgehog signaling pathway in cancer: A comprehensive review. Bosn. J. Basic Med. Sci..

[11] Heussler H.S., Suri M., Young I.D., Muenke M. (2002). Extreme variability of expression of a Sonic Hedgehog mutation: Attention difficulties and holoprosencephaly. Arch. Dis. Child..

[12] Jing D., Li C., Yao K., Xie X., Wang P., Zhao H., Feng J.Q., Zhao Z., Wu Y., Wang J. (2021). The vital role of Gli1^+^ mesenchymal stem cells in tissue development and homeostasis. J. Cell. Physiol..

[13] Hoashi T., Kanda N., Saeki H. (2022). Molecular Mechanisms and Targeted Therapies of Advanced Basal Cell Carcinoma. Int. J. Mol. Sci..

[14] Shivapriya P.M., Singh A., Pandey P., Chhabra N., Sahoo A.K., Paital B., Varadwaj P.K., Samanta S.K. (2021). Pathways in small cell lung cancer and its therapeutic perspectives. Front. Biosci. Landmark Ed..

[15] Iriana S., Asha K., Repak M., Sharma-Walia N. (2021). Hedgehog Signaling: Implications in Cancers and Viral Infections. Int. J. Mol. Sci..

[16] Riobo-Del Galdo N.A., Lara Montero A., Wertheimer E.V. (2019). Role of Hedgehog Signaling in Breast Cancer: Pathogenesis and Therapeutics. Cells.

[17] Jeng K.S., Sheen I.S., Leu C.M., Tseng P.H., Chang C.F. (2020). The Role of Smoothened in Cancer. Int. J. Mol. Sci..

[18] Liu J., Yin J., Chen P., Liu D., He W., Li Y., Li M., Fu X., Zeng G., Guo Y. (2021). Smoothened inhibition leads to decreased cell proliferation and suppressed tissue fibrosis in the development of benign prostatic hyperplasia. Cell Death Discov..

[19] Raleigh D.R., Reiter J.F. (2019). Misactivation of Hedgehog signaling causes inherited and sporadic cancers. J. Clin. Investig..

[20] Bhateja P., Cherian M., Majumder S., Ramaswamy B. (2019). The Hedgehog Signaling Pathway: A Viable Target in Breast Cancer?. Cancers.

[21] Dormoy V., Danilin S., Lindner V., Thomas L., Rothhut S., Coquard C., Helwig J.J., Jacqmin D., Lang H., Massfelder T. (2009). The sonic hedgehog signaling pathway is reactivated in human renal cell carcinoma and plays orchestral role in tumor growth. Mol. Cancer.

[22] D’Amato C., Rosa R., Marciano R., D’Amato V., Formisano L., Nappi L., Raimondo L., Di Mauro C., Servetto A., Fulciniti F. (2014). Inhibition of Hedgehog signalling by NVP-LDE225 (Erismodegib) interferes with growth and invasion of human renal cell carcinoma cells. Br. J. Cancer.

[23] Kotulak-Chrzaszcz A., Rybarczyk A., Klacz J., Matuszewski M., Kmiec Z., Wierzbicki P.M. (2022). Expression levels of sonic hedgehog pathway genes and their targets are upregulated in early clear cell renal cell carcinoma. Int. J. Mol. Med..

[24] Kotulak-Chrzaszcz A., Klacz J., Matuszewski M., Kmiec Z., Wierzbicki P.M. (2019). Expression of the Sonic Hedgehog pathway components in clear cell renal cell carcinoma. Oncol. Lett..

[25] Zhou J., Zhu G., Huang J., Li L., Du Y., Gao Y., Wu D., Wang X., Hsieh J.T., He D. (2016). Non-canonical GLI1/2 activation by PI3K/AKT signaling in renal cell carcinoma: A novel potential therapeutic target. Cancer Lett..

[26] Ding J., Li H.Y., Zhang L., Zhou Y., Wu J. (2021). Hedgehog Signaling, a Critical Pathway Governing the Development and Progression of Hepatocellular Carcinoma. Cells.

[27] Kim H.S., Kim Y.S., Lee C., Shin M.S., Kim J.W., Jang B.G. (2019). Expression profile of sonic hedgehog signaling-related molecules in basal cell carcinoma. PLoS ONE.

[28] Qi X., Schmiege P., Coutavas E., Wang J., Li X. (2018). Structures of human Patched and its complex with native palmitoylated sonic hedgehog. Nature.

[29] Xu Z., Liu Y., Yang Y., Wang J., Zhang G., Liu Z., Fu H., Wang Z., Liu H., Xu J. (2017). High expression of Mucin13 associates with grimmer postoperative prognosis of patients with non-metastatic clear-cell renal cell carcinoma. Oncotarget.

[30] Wang J., Mook R.A., Lu J., Gooden D.M., Ribeiro A., Guo A., Barak L.S., Lyerly H.K., Chen W. (2012). Identification of a novel Smoothened antagonist that potently suppresses Hedgehog signaling. Bioorg. Med. Chem..

[31] Sabol M., Trnski D., Musani V., Ozretic P., Levanat S. (2018). Role of GLI Transcription Factors in Pathogenesis and Their Potential as New Therapeutic Targets. Int. J. Mol. Sci..

[32] Kelam N., Racetin A., Polovic M., Benzon B., Ogorevc M., Vukojevic K., Glavina Durdov M., Dunatov Huljev A., Kuzmic Prusac I., Caric D. (2022). Aberrations in FGFR1, FGFR2, and RIP5 Expression in Human Congenital Anomalies of the Kidney and Urinary Tract (CAKUT). Int. J. Mol. Sci..

[33] Pastar V., Lozic M., Kelam N., Filipovic N., Bernard B., Katsuyama Y., Vukojevic K. (2021). Connexin Expression Is Altered in Liver Development of Yotari (dab1 -/-) Mice. Int. J. Mol. Sci..

[34] Cicchetti D. (1994). Guidelines, Criteria, and Rules of Thumb for Evaluating Normed and Standardized Assessment Instrument in Psychology. Psychol. Assess..

[35] Hammer Ø., Harper D., Ryan P. (2001). PAST: Paleontological Statistics Software Package for Education and Data Analysis. Palaeontol. Electron..

